# The Disintegration Process in Microcrystalline Cellulose Based Tablets, Part 1: Influence of Temperature, Porosity and Superdisintegrants

**DOI:** 10.1002/jps.24544

**Published:** 2015-06-12

**Authors:** Samy Yassin, Daniel J Goodwin, Andrew Anderson, Juraj Sibik, D Ian Wilson, Lynn F Gladden, J Axel Zeitler

**Affiliations:** 1 Department of Chemical Engineering and Biotechnology, University of CambridgePembroke Street, Cambridge, CB2 3RA, UK; 2 GlaxoSmithKline, New Frontiers Science ParkHarlow, Essex, CM19 5AW, UK

**Keywords:** terahertz pulsed imaging, structure–transport relationship, porosity, swelling, solid dosage forms, polymeric drug delivery systems, formulation, superdisintegrants

## Abstract

Disintegration performance was measured by analysing both water ingress and tablet swelling of pure microcrystalline cellulose (MCC) and in mixture with croscarmellose sodium using terahertz pulsed imaging (TPI). Tablets made from pure MCC with porosities of 10% and 15% showed similar swelling and transport kinetics: within the first 15 s, tablets had swollen by up to 33% of their original thickness and water had fully penetrated the tablet following Darcy flow kinetics. In contrast, MCC tablets with a porosity of 5% exhibited much slower transport kinetics, with swelling to only 17% of their original thickness and full water penetration reached after 100 s, dominated by case II transport kinetics. The effect of adding superdisintegrant to the formulation and varying the temperature of the dissolution medium between 20°C and 37°C on the swelling and transport process was quantified. We have demonstrated that TPI can be used to non-invasively analyse the complex disintegration kinetics of formulations that take place on timescales of seconds and is a promising tool to better understand the effect of dosage form microstructure on its performance. By relating immediate-release formulations to mathematical models used to describe controlled release formulations, it becomes possible to use this data for formulation design. © 2015 The Authors. *Journal of Pharmaceutical Sciences* published by Wiley Periodicals, Inc. and the American Pharmacists Association J Pharm Sci 104:3440–3450, 2015

## INTRODUCTION

In order to ensure a drug is readily available for absorption in as short a time as possible, the formulation design of immediate-release oral tablets is focussed on achieving rapid disintegration and dissolution. Immediate-release tablets are designed to fully disintegrate within a set period, normally within 2.5 min and not longer than 10 min.^1^,^2^ This can be particularly important where a rapid onset of action is required, for example for analgesics,^3^ or to achieve suitable bioavailability of a poorly soluble drug substance, that is to maximise dissolution by increasing the contact time with body fluids.^1^ In these cases, the formulation can be a critical factor in meeting the quality target product profile. Immediate-release formulations typically employ hydrophilic and hygroscopic excipients that facilitate disintegration by promoting media ingress and swelling of the tablet. Disintegrants are obviously important components of the formulation to establish these rapid release properties, however, other excipients may have an impact. The most widely used superdisintegrants are croscarmellose sodium (CCS), sodium starch glycolate and crospovidone. Relatively small amounts of superdisintegrants are added to tablet formulations, typically between 2% and 5% by weight,^3^ which is enough to influence the disintegration performance in the drug delivery systems via liquid transport enhancement by rapid swelling as well as gel formation.^4^–^6^

Microcrystalline cellulose^7^ (MCC) is a widely used flowability aid and diluent that can be pre-granulated or directly compacted.^8^ MCC is highly hygroscopic.^9^,^10^ In addition to the effect of disintegrants, MCC also enhances liquid transport into a tablet matrix, accelerating both diffusion and capillary action.^11^

The choice of superdisintegrant may be based on demonstration of compatibility with the drug substance and prior familiarity rather than a rational selection based on achieving optimum disintegration for the system under development.^5^ Other excipients such as lactose, mannitol, magnesium stearate and silica amongst other compounds are commonly added to the formulation to produce dosage forms which achieve desirable tablet properties in terms of fill weight and flowability.^12^–^15^

The chemistry of the excipients strongly affects disintegration performance, but other environmental and microstructural factors must be considered^16^: the tortuosity, porosity and pore size distribution will impact the rate of penetration of the dissolution solvent as well as the initial release of the active pharmaceutical ingredient (API).^16^ The hydration times are of critical importance in controlling the rate of disintegration of immediate-release formulations. An estimate of tablet porosity can be made based on calculation of the volume of a tablet and the weighted average of the true density of the formulation components. Alternatively, more sophisticated methods to measure the porosity of tablets directly are using liquid or gas intrusion.^10^,^17^

The analysis of immediate-release formulations before and during the disintegration process is essential in understanding drug release mechanisms and improving the efficiency of drug release.

It is well known that the porosity of tablets directly affects the rate at which water and other bodily fluids enter and propagate through the tablet matrix.^16^ In addition to the standard porosimetry techniques, we have recently demonstrated that the bulk porosity of the tablets can be measured using terahertz time-domain spectroscopy (THz-TDS).^18^,^19^ If the thickness of the tablet is known, the technique can be used to extract the porosity of the tablet following a fast non-invasive transmission measurement.^18^ X-ray microcomputed tomography can also be used to extract the porosity of the tablets, but is limited by the pixel size of the image to pores of approximately >2 μm.^20^ The porosity is of importance in predicting how liquids penetrate into the tablet matrix and the predictions can be validated by experimental analysis of solvent penetration.^21^ One typical method to resolve the penetration process is the use of magnetic resonance imaging (MRI).^22^–^26^ Hydration of tablets is measured by mapping the position of ^1^H-nuclei associated with mobile water, thus mapping out the ingress of water into the tablet matrix non-destructively. We have recently demonstrated that terahertz pulsed imaging (TPI) can also be used in this context.^27^ This method exploits the transparency of polymers at terahertz frequencies as well as the differences between the refractive index of polymers and solvents. Solvents typically have a higher refractive index and this change of refractive index provides the contrast between the solvent and polymer^28^ that can be used to track the water front into the tablet matrix.^28^

Traditionally, dissolution and disintegration performance is based on the release of API into the dissolution medium. Typical techniques which have been used to analyse the release of API, include UV–Vis/HPLC,^29^,^30^ MRI,^22^–^26^ thermo gravimetric analysis,^31^,^32^ infrared spectroscopy and infrared imaging^33^–^36^ amongst other techniques. These techniques have been applied to controlled release formulations to understand the release kinetics of the API and the tablet swelling kinetics,^37^ as well as to provide insight into how tablet formulation, geometries and dissolution kinetics affect API release during formulation development.

The types of transport which significantly contribute to the solvent transport into tablet matrices are capillary action and diffusion.^1^,^38^ Diffusion theory is used to directly relate swelling and transport in these systems^37^ by modelling either Fickian transport, which is driven by the concentration gradient across the tablet,^37^ and case II transport, which is solvent penetration dependent on an activity gradient such as swelling.^39^ Diffusion theory has been used to relate swelling and solvent ingress into the tablet and thus understand tablet dissolution performance.^25^,^39^–^41^ A common method of linking the swelling to the API release is by using a power law.


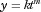
(1)

where *y* is the displacement of the API through the tablet matrix, *k* is a fitting parameter, *t* is the time and *m* is the power law constant that is characteristic of the transport processes observed.^37^ This value is dependent on the geometry of the tablet.^37^ For cylindrical tablets, *m* values of approximately 0.5 correspond to Fickian transport and values of approximately 1.0 relate to case II transport. This is a very simplistic transport model, which relates the velocity front of a solvent within the tablet to transport kinetics. More complex models such as the Higuchi model introduce concentration terms.^42^ Models have been developed and are used to describe API release in particular in controlled release formulations. The power law is one of the most commonly used mathematical models to describe API release in controlled release systems.

When considering transport in porous media, a major transport mechanism which should be considered is flow controlled by a gradient in capillary pressure throughout the tablet system, typically referred to as Darcy flow.^43^ A commonly used solution to describe Darcy flow behaviour is referred to as the Washburn equation (see also Supplementary Information)^44^


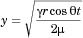
(2)

where *y* is the displacement of the solvent propagating through the solvent, *γ* is the surface tension, *θ* is the contact angle, *t* is the penetration time and *μ* is the dynamic viscosity.^45^ Work has been carried out to relate the Washburn equation with swelling to understand how the two processes interlink.^46^–^48^ In this study, we will use Darcy flow to model the liquid ingress into the porous media. It is better suited in this context compared with Fickian diffusion models as there is no inherent concentration gradient present within the pores when describing immediate release formulations where solvent penetration is studied into dry tablets and there is almost exclusively air occupying the pore space within the tablet.

Here, we use TPI to analyse the ingress of water into MCC-based immediate-release formulations, by taking advantage of the high time resolution of this technique and then applying the mathematical models discussed by Siepmann and Peppas^37^ and Costa and Sousa Lobo,^42^ normally applied to controlled release formulations, to immediate-release formulation, in order to resolve the impact of different excipients on the disintegration performance of immediate-release formulations. The formulations assessed in this study represent simplified systems, which were prepared to closely resemble commercial formulations, to provide an insight into the key characteristics during water ingress and disintegration.

## MATERIALS AND METHODS

### Materials

The samples analysed in this study were flat-faced tablet compacts made from 100% wt MCC (Avicel® PH-102; FMC BioPolymers, Philadelphia, Pennsylvania), a commercial grade of MCC that is typically used in direct compression formulations. In addition, tablet compacts containing both MCC and CCS (Ac-Di-Sol®; FMC BioPolymers) were prepared. CCS is one of the most widely used commercial superdisintegrants. Tablets were compressed to a thickness of 1.5 mm and diameter of 10 mm, with nominal calculated porosities of 0.05, 0.10, 0.15 and 0.40. Three tablet formulations will be discussed in this paper; 100% wt MCC, MCC 95% wt and CCS 5% as well as MCC 98% wt and CCS 2%. No lubricant was added to the formulation or applied directly to the punches/die during compression.

### Porosity Measurements

Porosity measurements were carried using a THz-TD spectrometer in transmission mode on pure MCC tablets, using the setup described by Parrot et al.^49^. The beam waist at the focus of the tablet is 6 mm in diameter and therefore porosity measurements using THz-TDS represent the central 36% of the tablet volume. Using the technique described by Bawuah et al.^18^, the refractive index (*n_t_*) of the tablets is extracted and from this the permittivity (*ε*_eff_) of the tablets can be calculated, where *ε*_eff_ = *n_t_*^2^. The method is calibrated by two known refractive indices at known porosities. Given the relative permittivity of air (*n*_a_ = 1, *ε*_a_ = 1), it is possible to calculate the relative permittivity of MCC (*ε*_b_):


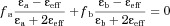
(3)

where *ε*_eff_ is the permittivity of the tablet, *f*_a_ is the fraction of the tablet taken up by air (void fraction) and *f*_b_ is the fraction of the tablet taken up by MCC (solid fraction). The relative permittivity of MCC can then be used to calculate the porosities of MCC tablets with different porosities using the obtained refractive indices.


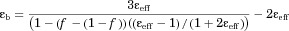
(4)

Equation 4 is solved for 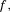 which is the tablet porosity. Density measurements of the solid matrix of the tablets were obtained using the Accupyc 1330 Pycnometer (Micrometrics, Norcross, Georgia). Initially samples were weighed and then placed into a 1 cm^3^ cup. The sample chamber was pressurised to 10 bar with helium and this was repeated 30 times, after which a density reading for the solid matrix was recorded (solid density *D*_p_). Using these data the density of the void space within the tablet was determined and the porosity was calculated. These values were then used as known porosities to compare with the terahertz time-domain measurements, as well as an input for the known tablet porosity *f*_b_.

Porosities were also calculated by dividing the mass of the tablet by its volume, resulting in bulk density (*ρ*_b_) of the tablet (*ρ*_b_ = *m*/*v*). The bulk density is then divided by the weighted average of the true density of each excipient within the formulation (*ρ*_t_) to produce the solid fraction of the tablet, subtracting this value from unity will then result in the total porosity of the tablet (*f*).

### Compression of Tablet Compacts

**Figure 1:**
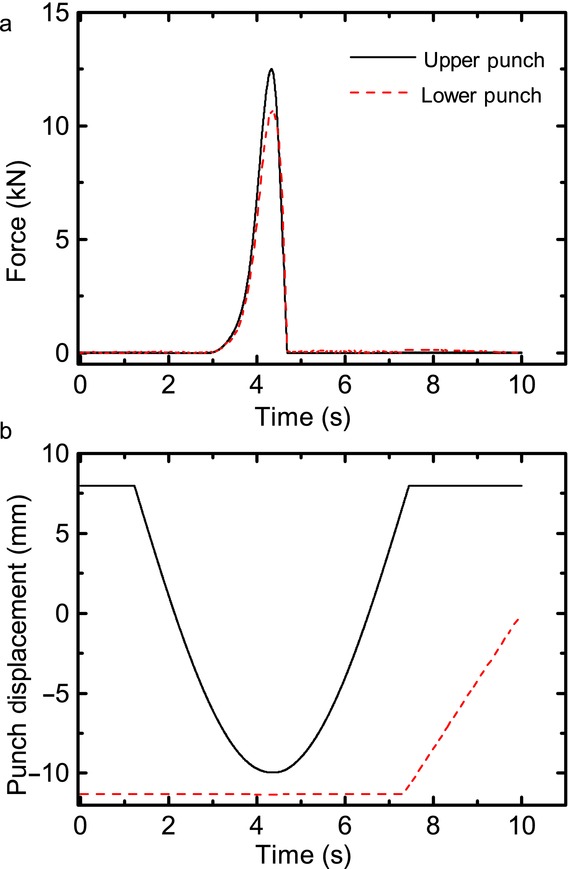
Typical compaction profile measured by the compaction simulator, where (a) is the compaction force profile of both the upper and lower punch and (b) is the punch displacement. This is an example of a profile producing a pure MCC tablet of 10% porosity.

Flat-faced, round tablet compacts of 10 mm diameter were compressed using a compaction simulator (Phoenix Calibrations, Phoenix, Arizona). The device was instrumented such that it can measure the forces of compaction throughout the compression event. Powder was loaded into the dies using a hopper and volumetric filling was performed to ensure tablets were achieved with target porosities of 5%, 10% and 15%. A secondary method of die fill was also studied where the die was manually filled, producing pure MCC tablets with 10% and 40% porosities. Tablets were compressed over a 10 s period using a uniaxial compression profile, Figure 1.

### Disintegration Testing

Disintegration tests were carried out using a disintegration tester (DT) (Agilent Technologies, Santa Clara, California). Tablets are hydrated using water at 37°C, and experiments are carried out and timed until tablets fully disintegrate (no visible residue of the tablet remain on the screen of the test apparatus or adhere to the lower surface of the discs).

### Transport Analysis using TPI

**Figure 2:**
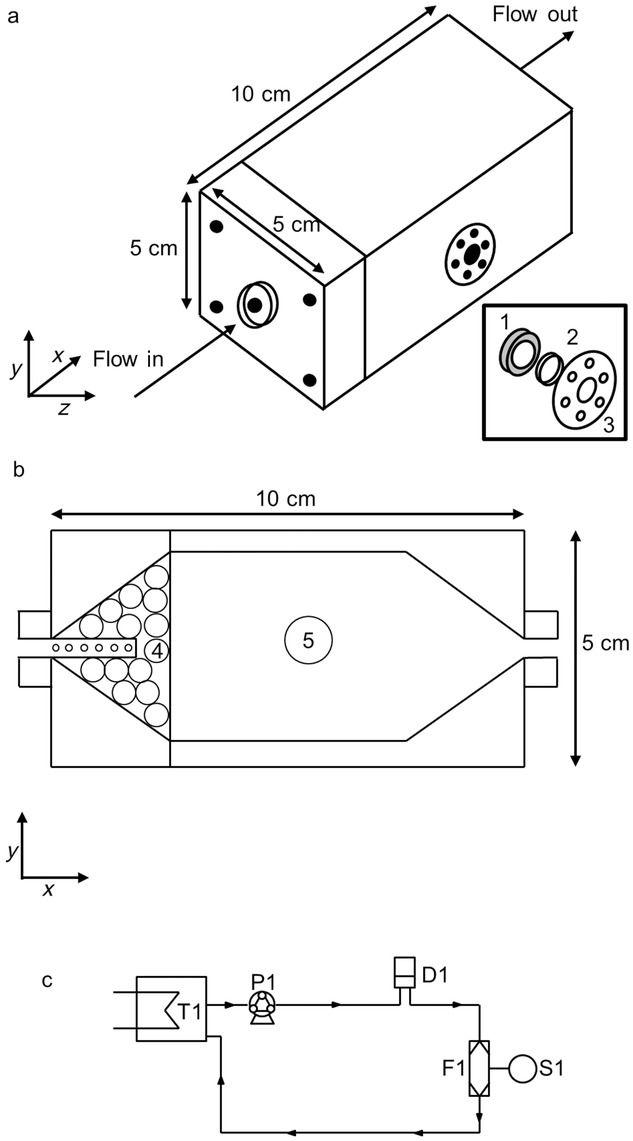
Newly developed flow cell, where panel (a) shows the exterior of the flow cell and panel (b) shows an internal cross-section at the centre of the cell, showing the flow geometry. 1 is the sample holder with a sealant to ensure only one-dimensional transport through the axial surface of the tablet occurs, 2 is the tablet sample, 3 is a polyethylene window, 4 is a bed of 0.5 cm glass ballotini beads and 5 marks the position of the tablet in the flow cell. Panel (c) is the schematic representation of the flow loop; T1 the water tank and heat exchanger, P1 the peristaltic pump, D1 is a flow dampener, F1 is the flow cell and S1 is TPI sensor.

Transport and swelling kinetics were measured using a modified TPI imaga 2000 (Teraview Ltd., Cambridge, UK) as outlined previously in more detail by Yassin et al.^27^. In this study, a flow cell design was developed to achieve more reproducible measurements (Fig. 2).

**Figure 3:**
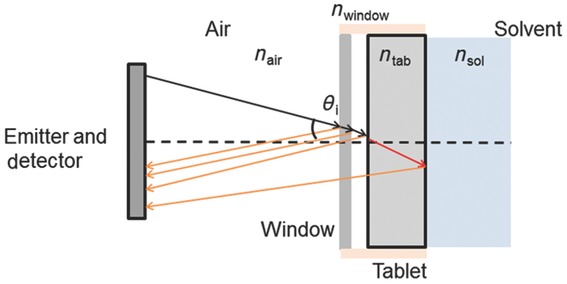
Schematic diagram describing how terahertz radiation interacts with the experimental setup and sample. *n*_air_ is the refractive index of air, *n*_tab_ is the average refractive index of the tablet and *n*_sol_ is the refractive index of water.

The cell was developed to ensure that water only contacts tablets from a single axial surface (this will be referred to as the back surface in the remainder of the paper); hence, this technique is assessing the one-dimensional transport of water into the tablet matrix. The water flow was set to 140 mL/min. The cell was designed such that the front surface of the tablet sits at a distance of 7 mm from the terahertz optics, which is the focal length of the TPI Imaga 2000 optics (Fig. 3). In our experiments, 15 measurements are being acquired per second. In order to remove noise, five waveforms are co-averaged resulting in an effective sampling rate of three waveforms per second.

The TPI measurements can resolve the ingress of water as it enters the tablet matrix and the subsequent swelling of the matrix itself. The way in which water transport is measured is by exploiting the transparency of the polymers to terahertz radiation and monitoring the changes in refractive index between the tablet and water. Initially, the tablet has a refractive index between that of the solid matrix material and air (*n* = 1.0), which is occupying the pores of the material as described in Eq. 3. As water starts to wet the tablet matrix, it displaces the air in the pores and increases the refractive index of the material, water having a higher refractive index (*n* = 2.4)^50^ than the solid matrix. This change in refractive index provides contrast, which makes it possible to monitor the ingress of the water into the tablet. Each time the terahertz pulse encounters a change in refractive index a portion of the pulse will be reflected back to the emitter.

The analysis of the results follows the method outlined by Yassin et al.^27^, with a modification to account for the changes due to the new flow cell as an additional reflection from the newly introduced PE window needs to be considered during the analysis. To ensure all artefacts are removed, an additional averaging step is introduced. Each full waveform contains 2048 points. The window of interest typically spans from points 600 to points 1800, which corresponds to an optical time delay of 0–3.5 mm in air. Box widths of 50 points were averaged on each waveform (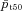) and each of these boxes are averaged over the total experiment (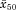). This value is then subtracted from each point on the waveform, at the region of interest (600–1800), on each individual waveform (*p*_t_). This process can be shown in Eq. 5 below:


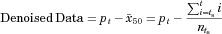
(5)

where (*t*) is the experimental run time, (*t*_s_) is the time between each time point and (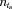) is the number of time steps.

## RESULTS AND DISCUSSION

### Demonstration of the New Flow System

**Figure 4:**
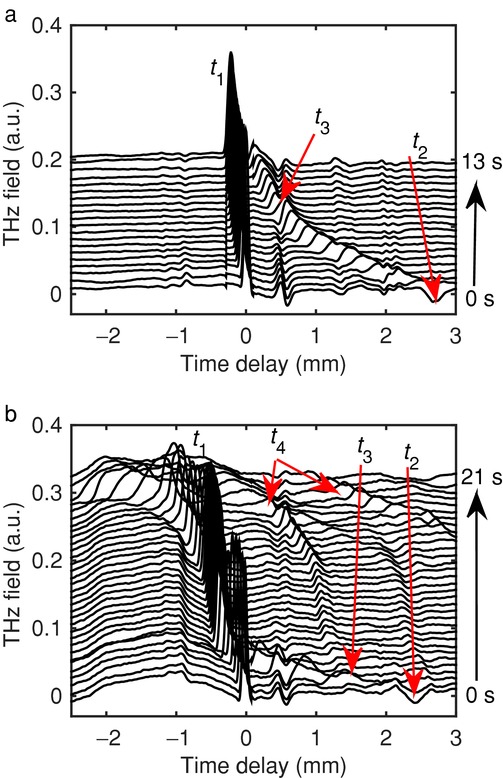
(a) Deconvolved time-domain waveforms showing the hydration of a MCC tablet of 10% porosity (each line is offset by 0.01 a.u.); and (b) 40% porosity MCC tablet (each line is offset by 0.005 a.u.). *t*_1_ corresponds to the front surface of the tablet, *t*_2_ corresponds to the back surface of the tablet, *t*_3_ corresponds to the hydration front and *t*_4_ corresponds to the formation of cracks within the tablet matrix due to the strain caused by the hydration.

For a proof-of-principle experiment samples of 100% wt MCC with porosities of approximately 10 ± 5% and 40 ± 5% were prepared by direct compression following manual filling of the die without hopper. Waterfall plots were produced to visualise the transformations qualitatively in each tablet over the experimental run time. Two examples are shown in Figure 4.

Liquid ingress is apparent in both samples (*t*_3_ front highlighted in red). It is clear that in the 40% porosity sample, the transport of water through the tablet matrix is much faster: the penetration of water through the entire tablet takes about 3 s as opposed to approximately 13 s in the 10% porosity tablets of the same thickness. An interesting result is observed in that rather than a single peak multiple penetration fronts are observed in the tablet of 40% porosity. This is caused by the simultaneous collapse in structure during the hydration process, leading to cracks on both the tablet surface and within the tablet matrix that can be detected by the additional reflections. It also leads to larger volumes of water being able to enter the tablet matrix.

**Figure 5:**
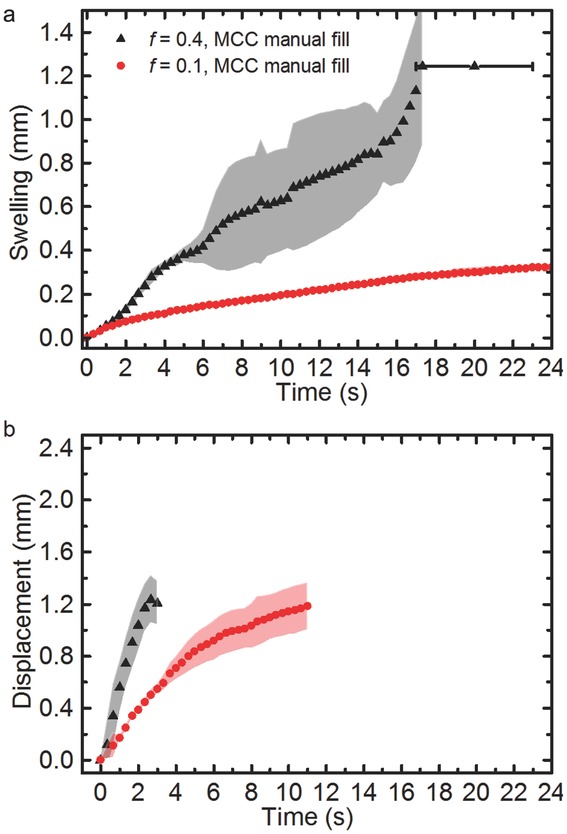
(a) Average swelling profile of tablet samples of pure MCC of 10% porosity (red circles) and 40% porosity (black triangles) at a water temperature of 20°C. (b) Corresponding transport kinetics based on the position of the water front within the pure MCC expressed as the displacement of the penetration front relative to the back surface of the tablet. The shading marks the SD (*n* = 3). The error bar represents the average disintegration time and SD (*n* = 3). The tablets with 10% porosity do not disintegrate.

**Figure 6:**
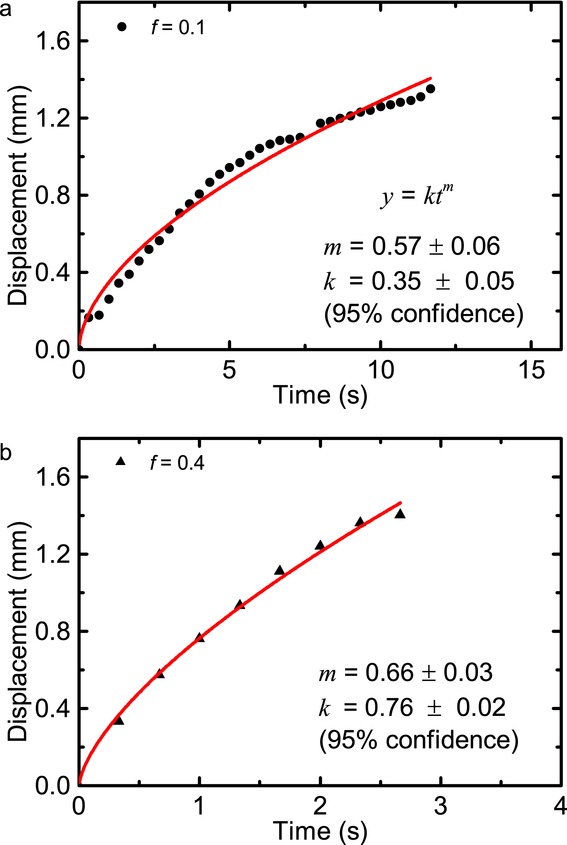
Experimental transport kinetics fitted using the power law, where black dots represent data obtained experimentally and the red lines are the power law fit. (a) 10% porosity MCC tablet; and (b) 40% porosity tablets. Both samples were compacted following manual filling of the dies.

Swelling is observed in both the 10% and 40% porosity tablets (Fig. 5a). Not only do the 40% porosity tablets start to fracture internally upon water ingress but they also undergo strong expansion, swelling by up to 1.5 mm at the front surface. The tablets typically break apart after approximately 15 s. In contrast, the 10% porosity MCC tablets expand by only 0.2 mm at the front surface. They largely retain their shape throughout the experimental procedure and do not break within 200 s. This can be explained by the difference in magnitude of the applied compaction forces that act on the MCC powder during the tableting process: to produce tablets with 10% porosity, approximately 7.0 kN of force are applied, whereas only 0.4 kN are applied to achieve 40% porosity. The 40% porosity tablets represent an extreme system, which would not be used in final formulations, but it does provide an insight into how large porosities can impact tablet formulations. It is well known that the strength of the sinter bridges formed between the particles are controlled by the compaction force, where a higher compaction force produces stronger sinter bridges.^51^ As the MCC is being hydrated some particles begin to adsorb water given MCC's hygroscopicity. This process, which is independent of temperature,^52^ weakens the interparticular bonds and results in swelling of the tablet. This swelling together with the relatively large volume of water available in the 40% porosity sample facilitates breaking of the sinter bridges. A secondary process which further advances swelling is the slow relaxation of the axial stress due to the compaction process, originating from the elastic deformation of the MCC powders within the tablet matrix.^53^ This relaxation will be accelerated during hydration.^54^ The relative strength of the intraparticular bonds affects the time it takes for the tablet to fully disintegrate: tablets with a porosity of 40% break down in 18 ± 5 s, whereas the 10% porosity tablets do not disintegrate over the timescales of the experiment. It is clear that towards the end of the swelling process in the 40% porosity tablets that the reproducibility becomes poor and this is because the individual tablets are breaking at different time points. The rate of swelling affects the penetration kinetics (Fig. 5b). It is clear that in the 40% porosity tablets there is a more linear profile in water transport into the tablet compared with that of the 10% porosity tablets. This process can be quantified by applying the power law Eq. 1 to the position of the water front over time Figure 6.

### Effect of Porosity and Temperature on MCC Disintegration

In order to investigate the disintegration process in more detail, a second set of MCC samples was prepared with total porosities spanning a smaller range (5%, 10% and 15%). In addition, the measurements were performed at two water temperatures (20°C and 37°C) to further explore the effect of the dissolution medium on the disintegration process. Prior to the TPI measurements the bulk porosities of the samples were determined using helium pycometry and THz-TDS (Table1).

**Table 1:** Porosity Measurements of the MCC Tablets Obtained using Helium Pycnometry and THz-TDS

Sample	*ψ* (cm^3^)	*f*_p_	*f*_THz_	*f*_calc_
5P1	0.116	0.045	N/A	0.058
5P2	0.116	0.042	N/A	0.058
10P1	0.107	0.100	0.103	0.106
10P2	0.106	0.109	0.105	0.104
15P1	0.104	0.141	0.148	0.157
15P2	0.104	0.144	0.142	0.152

*Ψ* is the solid volume calculated using helium pycnometry (the SD between 10 repeat measurements for each individual sample was better than 0.001 cm^3^ in all samples tested); *f*_p_ is the resulting porosity calculated based on the volume measured by pycnometry and the tablet mass; *f*_THz_ is the porosity calculated using THz-TDS based on the sample thickness and refractive index; and *f*_calc_ is the calculated porosity of each sample as outlined in the introduction. 5P refers to samples of 5% nominal porosity, 10P to 10% nominal porosity and 15P to 15% nominal porosity.

**Figure 7:**
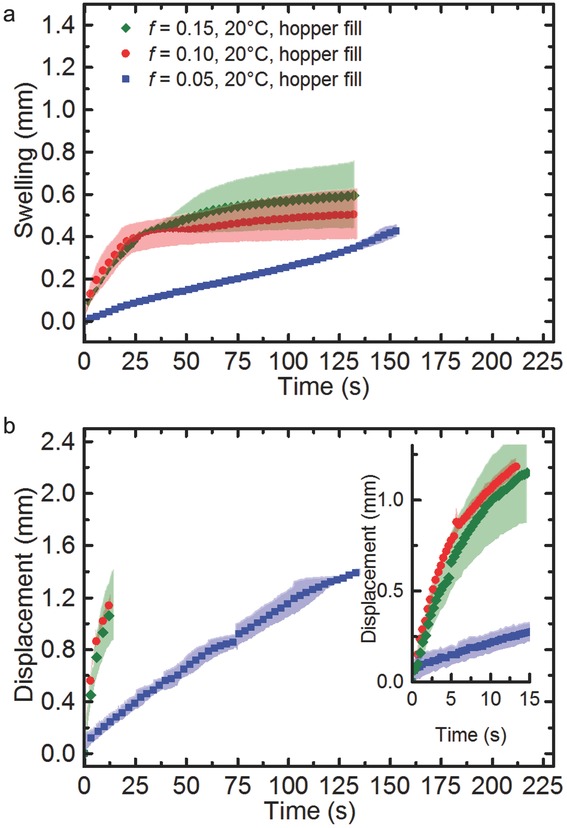
(a) Swelling and (b) transport kinetics, in pure MCC tablets at a water temperature of 20°C. The background shading corresponds to the SD between three, five and three tablets, respectively, for the samples of 15%, 10% and 5% porosity, respectively. For clarity only every 10th data point is plotted except for the inset of (b) where all data points are shown. The tablets do not disintegrate over the duration of the experiment.

The reproducibility of the results, in particular regarding the movement of the penetration fronts of water into the sample, is improved in this second set of samples compared to the previous set when assessing tablets compacted to 10% porosity (Fig. 7): there is less variation in the swelling behaviour which results in a lower SD between the measurements. The only difference between the samples is the method used to fill the die with MCC powder prior to compaction. The initial samples were prepared by manual die filling, whereas the second set of samples were prepared using automated hopper filling of the die. It is interesting to note that it appears that the manual filling process caused sufficient variation in the microstructure of the tablets that caused a significant change in the penetration kinetics.

When comparing the three different sets of samples, the rate of swelling and solvent penetration was found to correlate with the porosity of the tablets. Transport of water from the back surface of the tablet to its front in the 15% porosity tablets occurs on average within 19 s. In both the 15% and 10% porosity tablets, rapid swelling of the tablet matrix is observed within 30 s. During this period, the tablet matrix expands by up to 50% of its original thickness and full liquid penetration is achieved. The tablet expansion continues more gradually for the remainder of the experiment. The rate of swelling reduces after approximately 25 s for both the 10% and 15% porosity tablets (Fig. 7). This change of rate can be attributed to the saturation of the MCC particles with water within the tablet matrix. Upon saturation, the rate of uptake of water begins to decrease resulting in a slowdown of the rate of swelling.^52^ Within the largely laminar flow regime that the tablets are exposed to at their back the samples remain intact throughout this process without fully disintegrating even following their complete hydration. In contrast, the 5% porosity tablets exhibit quite a different hydration behaviour. Overall the transport kinetics in the 5% porosity samples are slower and more linear. It takes on average 148 s for the water to propagate from the back surface of the tablet to its front. In terms of the swelling behaviour, two phases can be resolved: gradual swelling the first 125 s; this is then followed by a slightly more rapid expansion phase. The magnitude of swelling in the 5% porosity tablets is of similar magnitude to that of the higher porosity tablets. In the case of the 5%, 10% and 15% porosity tablets, the tablet matrix held its shape throughout the experiment.

**Figure 8:**
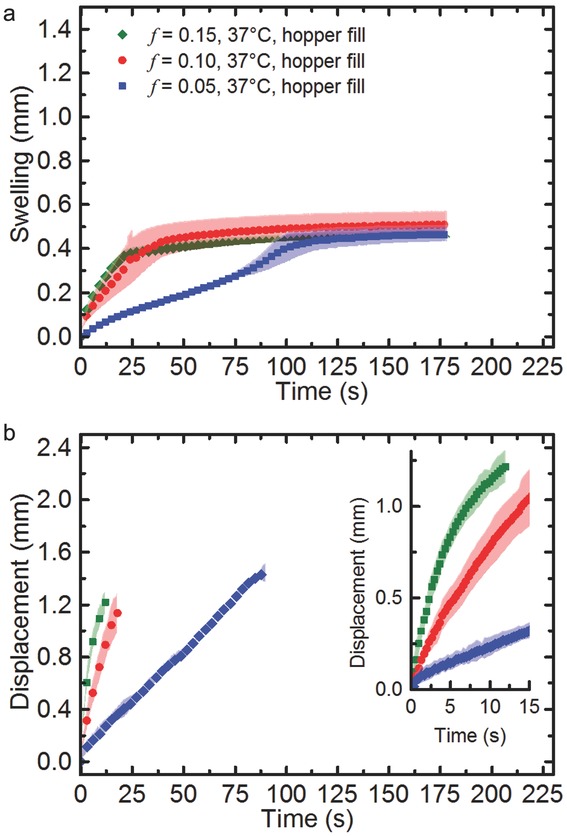
(a) Swelling kinetics and (b) transport kinetics in MCC at a water temperature of 37°C. The shading in the background of each plot corresponds to the SD between five, six and three tablets for the samples of 15%, 10% and 5% porosity, respectively. For clarity only every 10th data point is plotted except for the inset of (b) where all data points are shown. The tablets do not disintegrate over the duration of the experiment.

In order to investigate the influence of the water temperature on the disintegration process, the experiments have been repeated with warmer water at approximately body temperature (Fig. 8).

As the water temperature is increased to 37°C, the swelling and transport is consistently faster compared with a temperature of 20°C. This is not surprising as the kinetic energy in water increases with temperature. Overall the swelling profiles look similar in shape to the experiments carried out at the lower temperature except for the swelling behaviour in the 5% porosity tablets, which appears to be less linear at the higher water temperature. As was the case for the tablets measured at 20°C, the tablet matrices do not disintegrate during the experiment at 37°C.

The results from DT show no correlation between porosity and disintegration time: MCC tablets were found to disintegrate within 333 ± 30 s at 5% porosity, 597 ± 0 s at 10% porosity and 37 ± 3 s at 15% porosity. The results highlight that in the case of pure MCC tablets, the disintegration test, as outlined by the pharmacopoeias, is providing information on the mechanical stability of the tablets rather than their disintegration performance due to interaction with solvent. Here, the mechanical shear applied to the tablets as well as the tablet collisions with the wall of the DT lead to disintegration and it is the physical properties of the tablets such as plasticity, brittleness and elasticity which are being tested for rather than the changes in microstructure induced by wetting with solvent.

### Effect of Disintegrant on MCC Matrix Disintegration

**Figure 9:**
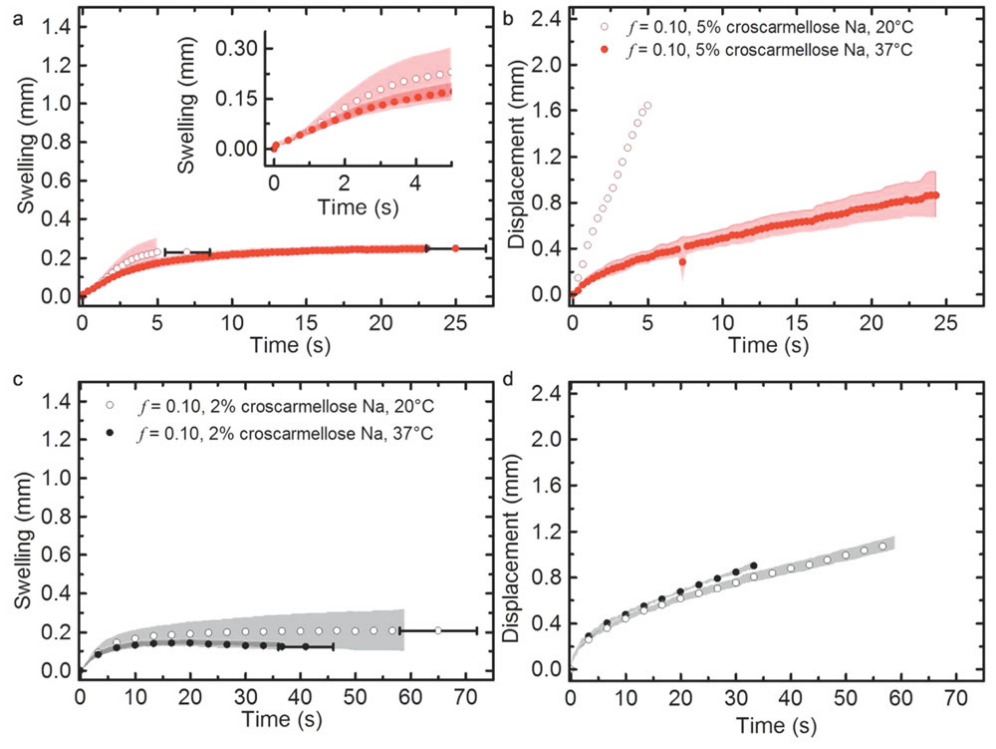
(a) Swelling kinetics and (b) corresponding transport kinetics in tablets of 10% porosity containing a formulation of 95% MCC and 5% croscarmellose. (c) Swelling kinetics and (d) transport kinetics of a formulation of 98% MCC and 2% CCS. The shading in the background marks the SD of five repeat experiments. The *x*-error bar represents the average disintegration time and its SD (*n* = 5) for samples containing 5% CCS and 2% CCS. For clarity only every 10th data point is plotted.

In order to investigate the effect of adding superdisintegrant to the formulation, samples of 95% MCC with 5% CCS and 98% MCC with 2% CCS were prepared. The tablets had an overall porosity of 10% and TPI disintegration testing was performed at 20°C and 37°C (Fig. 9).

By adding a superdisintegrant to the formulation, the tablet disintegration dynamics changed dramatically. In contrast to the pure MCC tablets with porosities of 5%, 10% and 15%, the tablets containing the disintegrant break down consistently and water transport into the tablet matrices is accelerated. At 20°C, liquid transport through the entire tablet takes place over a 6 s period in tablets containing 5% CCS. During this time, the tablets swell by up to 20% of their initial thickness and all tablets were found to break apart within a total of 7 s. When the temperature is increased to 37°C, the process slows down significantly. Liquid transport takes place on average over 38 s, accompanied by swelling to approximately 17% of its original thickness. Break down of the tablets is observed during the hydration process, in 6 ± 1 s at 20°C and within 38 ± 19 s at 37°C. When the concentration of the disintegrant is reduced to 2% there is a significant change in the tablet disintegration kinetics. At 20°C, liquid uptake slows down, which is reflected in an apparent change in transport mechanism and disintegration times fall to 65 ± 7 s. At temperatures of 37°C, the disintegration kinetics is similar to samples containing 5% CCS with total disintegration occurring within 41 ± 5 s.

As expected, the hydration process is consistently faster in the samples that contain CCS compared with the pure MCC tablets. This can be explained by its ability to swell up to 300% of its volume, which allows it to break apart the tablet when evenly mixed into the tablet matrix.^5^ In contrast to the plain MCC tablets, where faster hydration was observed at higher temperature, the increase in temperature lead to slower disintegration characteristics once CCS was added to the formulation. This can be explained by the fact that CCS forms a gel when in contact with water. Hydrogel formation is facilitated at increased temperatures^55^ and the disintegration process hence slows down upon hydration at 37°C. Upon gel formation, the gel immediately fills the macropores and interparticulate voids thus slowing down further water ingress as well acting as a binder because of the increased viscosity of the gel compared with the water. In addition the hydrogel acts as a barrier to further liquid ingress because of its hydrophobic nature, which also slows down the disintegration process.^55^ At 20°C hydrogel formation in CCS occurs at a lower rate resulting in much faster penetration of water into the tablet matrix. The samples containing 2% CCS exhibit slower disintegration kinetics compared to the samples containing 5% CCS. The reduced amount of CCS within the tablet decreases the effect from the swelling of the CCS particles. We have observed a marked change in disintegration kinetics as a result of a relatively small change in concentration of CCS and we believe this is a significant result given that a concentration of 0.5%–5% CCS is the typical range used in tablet formulations.

In the case of the tablets containing disintegrant, the DT results correlated well with the results obtained using the TPI at 37°C where tablets containing 5% CCS broke apart in 30 ± 4 s and tablets containing 2% CCS disintegrate in 26 ± 3 s. This can be explained by the fact that for the tablets containing disintegrant, the rapid swelling action of the disintegrant dominates the disintegration process, whereas the MCC alone does not disintegrate without external mechanical activation which is absent in the TPI measurements.

### Summary of the TPI Hydration Analysis

Typically, the pharmacopoeias require that the DT should be carried out at 37°C, yet our experiments show that there is a large difference in disintegration kinetics between 20°C and 37°C. Given that patients might swallow tablets with a cold cup of water (which will be far below 37°C and often even below 20°C), understanding tablet disintegration at temperatures other than 37°C is of key importance.

Using the power law equation introduced earlier, the transport kinetics of all the water transport properties of the tablets studied in this paper can be summarised by their exponent *m* (Fig. 0). The power law is widely used to describe both API release and liquid penetration into polymer systems by relating the position of the propagating component with time.^28^,^37^ The advantage of applying this simple model to the TPI data in order to resolve the transport kinetics is that it does not require the knowledge of the water concentration,^37^ opposed to both Fick's law of diffusion^56^ and the Higuchi model.^37^

**Figure:**
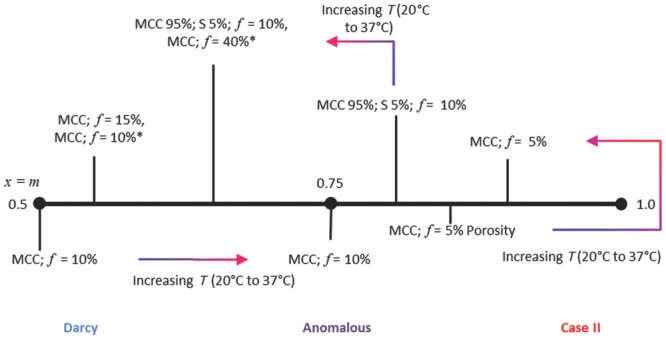
Schematic representation showing a classification of the type of transport observed in each sample studied based on the *m* values extracted from the power law fit of the respective transport kinetics measurements. Samples marked by * were prepared by manual die filling prior to compaction; all other samples were filled using a hopper. *f* refers to porosity.

As briefly discussed in the *Introduction*, Darcy's law appears to be the most suitable model to describe the initial water penetration process into these systems compared with the Fick's law of diffusion as there is no concentration gradient of water throughout the porous network in our samples. Here, it is assumed that a gradient in capillary action will be the gradient which regulates liquid penetration. A model of the liquid ingress has been derived (Eq. 6), which provides a solution to Darcy's law that mathematically follows the same form as the power law from Fick's diffusion.


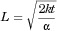
(6)

Here, *L* is the displacement of the liquid ingress front, *k* is the permeation rate constant, *t* is the time of displacement and *α* is the swelling ratio as derived in the attached Supplementary Information. This result shows that the liquid displacement in the Darcy flow regime will propagate as a function of the square root of time and hence can be approximated using a power law, *y* = *kt^m^*, when *m* = 0.5.

Using a fit of the power law, *y* = *kt^m^*, to the experimental data the exponent *m* can be determined, where values of 0.5 indicate Darcy flow, 0.75 anomalous transport and 1.0 case II transport.^41^ Darcy flow is dependent on a gradient of capillary pressure from low to high concentration.^39^ Case II transport is driven by an activity gradient, which is the effect of swelling on the transport of solvent into the tablet, due to inhibited transport from either increased tortuosity, the formation of hydrogels, reduced porosity of the tablet or enhanced swelling behaviour amongst other effects.^39^ These are standard physical phenomena which can be considered to cause case II transport.^39^ Anomalous transport shows both Darcy flow and case II characteristics.

The *m* values obtained for the 10% porosity samples ranged from 0.54 ± 0.02, 0.50 ± 0.04 and 0.73 ± 0.09 depending on how they were prepared and at what temperature the experiment was carried out. These values indicate that at the lower temperature Darcy flow dominates but when the temperature is increased to 37°C the transport mechanism becomes anomalous and therefore gains case II character: both the magnitude and the rate of swelling increase with temperature, which in turn accelerate the concomitant water transport resulting in transport kinetics that are dependent on both concentration and activity gradients.

For the samples of 5% total porosity, *m* values are found that correspond to anomalous transport with strong case II character. This is caused by the lack of sufficiently large (and connected) pores within the tablet matrices which severely hinders liquid transport. Therefore, the relative rate of swelling is greater in comparison with the rate of solvent penetration and thus the swelling process starts to govern the transport kinetics of the liquid ingress.

In contrast, in the 15% porosity tablets the large amount of pore space within the tablet matrices makes liquid transport very quick relative to the swelling and therefore Darcy flow is expected to dominate, which is confirmed by the measured *m* values. However, when the porosity is increased to 40%, swelling becomes so rapid that it significantly affects the liquid transport and hence swelling and water ingress occur on similar time scales. Consequently both concentration and activity gradients again affect the transport through the tablet. It is important to note in this context that the particle size of the formulation has a direct impact on the pore size distribution within the resulting tablet matrix after compaction, where small particles result in smaller pores and larger particles typically produces tablets with larger pore size distributions.^57^ Pore size distributions and porosity are also inversely proportional to the compaction force. This change in pore size distribution has a direct impact on the transport kinetics of the solvent through the tablet matrices with slower solvent transport in tablets of lower porosity and with smaller pore size.^1^,^58^ This effect can be resolved in the pure MCC samples in this study where the 5% porosity samples exhibited considerable slower transport kinetics compared with the higher porosity ones.

By adding superdisintegrant to the tablet matrices, both the swelling and transport kinetics change dramatically. At both temperatures swelling is significant but at the higher temperature the swelling process is considerably slowed down because of the increased gel formation, which results in Darcy dominated flow character. At temperatures below the gel formation the swelling is more rapid and hence case II characteristics dominate the mass transport. These observations correlate well with previous data.^55^

Case II kinetics is broadly defined as a transport process which is influenced by an activity gradient. We found that a number of different factors can result in such behaviour of a tablet formulation: (1) as a result of hindered transport because of the lack of available pore volume, for example as highlighted in the 5% porosity samples; (2) as a result of rapid swelling caused by the addition of a superdisintegrant such as CCS; or (3) because of the weaker compacts produced by the lower compaction forces as shown in the 40% porosity samples.

## CONCLUSIONS

Transport and swelling kinetics were measured in MCC over a range of porosities and multi component tablets of MCC and CCS. We have developed a method to quantitatively measure the magnitude of swelling and resolve the velocity front of water penetrating the tablet on timescales suitable for the analysis of rapid release formulations.

Using this setup, it was possible to investigate the effect of subtle differences in the compaction process on the disintegration performance as well as effects of temperature and porosity. Manual die filling showed a significant effect on the disintegration behaviour of the resulting tablets and this could be resolved using the TPI technique.

We showed that a number of different factors can drastically affect the disintegration behaviour of an immediate-release tablet formulation: (1) the available pore volume and its role in tablet hydration; (2) the effect of rapid swelling caused by the addition of a superdisintegrant such as CCS; (3) the strength of the interparticular sinter bridges; and (4) the temperature of the dissolution medium. Any one of these factors can easily double or halve the disintegration timescales. We believe that by taking into account these physicochemical characteristics it is possible to improve the formulation design for immediate-release tablets and achieve a more consistent and robust performance of such formulations over a wide range of conditions.

It is clear that TPI can be used to analyse immediate-release tablets in a manner which was previously possible only in controlled release tablets. Taking advantage of the high time resolution that can be achieved by TPI makes it possible to analyse liquid ingress, tablet swelling, microstructural changes and disintegration times quantitatively. This makes it possible to start resolving how different formulation components and microstructures affect the tablet disintegration and might allow for a more rational design of immediate-release formulations in the future.
