# Supplementary material for: The Disintegration Process in Microcrystalline Cellulose Based Tablets, Part 1: Influence of Temperature, Porosity and Superdisintegrants
Source: J Pharm Sci. 2015 Jun 12;104(10):3440–50. doi: 10.1002/jps.24544 (PMC4647644; doi:10.1002/jps.24544)
Supplement: Supplementary file 1 — Supplementary [file jps0104-3440-sd1.pdf]

Supplementary Information to

The Disintegration Process in Microcrystalline Cellulose Based Tablets I: Influence of Temperature, Porosity and Superdisintegrants

S. Yassin,[1] D.J. Goodwin,[2] A. Anderson,[2] J. Sibik,[1] D.I. Wilson,[1] L.F. Gladden,[1] and J.A. Zeitler[1,*]

[1] *Department of Chemical Engineering and Biotechnology, University of Cambridge, Pembroke Street, Cambridge CB2 3RA, UK*

[2] *GlaxoSmithKline, New Frontiers Science Park, Third Avenue, New Frontiers Science Park, Harlow CM19 5AW, Essex, UK*

(Dated: May 12, 2015)

In this supplementary section we briefly derive the mass transport model used to describe the water penetration into dry immediate release formulations based on microcrystalline cellulose and superdisintegrant.

In this work we have investigated the one-dimensional propagation of a liquid (water) front into a porous medium (flat faced tablet of compressed particles of microcrystalline cellulose, MCC, and other pharmaceutical excipients). In order to quantify the movement of the water front in the tablet from its back surface to the front we can model the process using Darcy flow characteristics. Darcy flow describes the flow of fluids through porous media and is widely used in chemical engineering and the earth sciences to express mass transfer in sand beds and rocks[1,2].

In the following derivation it is assumed that the cross-sectional area is unity and that the flow is laminar, which is a valid assumption given the measurement spot at the centre of the tablet in our experiments, which should make sure the measurements are not affected by boundary effects. We also restrict our analysis to the central volume element of a cylindrical tablet where water penetration occurs exclusively in the axial direction from a single face of its circular surface. There is no exposure of the cylindrical walls of the tablet to water and radial swelling is not possible due to the tablet being held tightly in a retaining ring throughout the hydration experiment. The geometry of the experiment is described in detail in the main document of this manuscript. Swelling will occur both in the direction of the flowing water region as well as towards the opposite direction at the same magnitude.

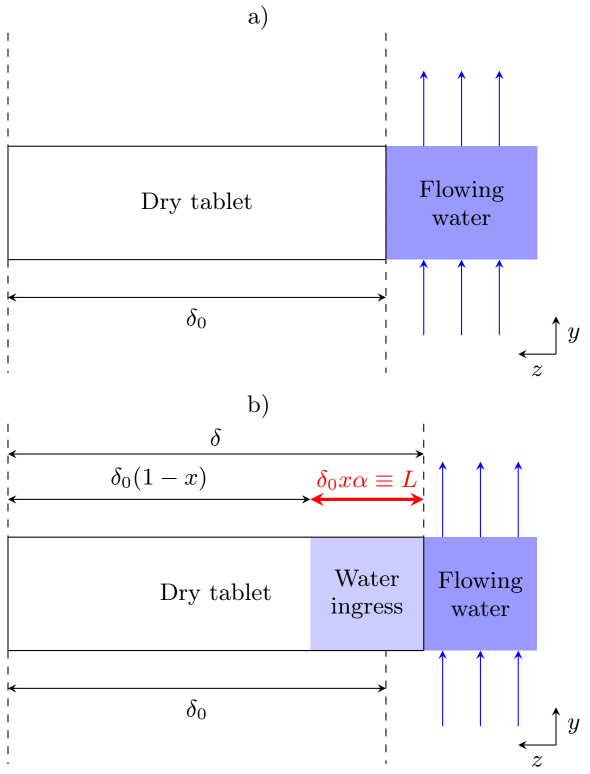

FIG. 1. Schematic of the liquid penetration model. The frame of reference is the front surface of the tablet that is not in contact with the liquid medium (left) as this is the first interface encountered by the THz pulse. a) Dry tablet at $t = 0$; and b) partially hydrated tablet.

I. DERIVATION

Consider a semi-infinite sheet of material of initial thickness δ_0 and voidage ε_0, as shown in Fig. 1a. On contact with water, water penetrates into the tablet, promoting swelling. Swelling is assumed to be instantaneous following contact of the biopolymer with water.

Let the fraction of material contacted with water be x: its dry thickness is $\delta_0(1-x)$ and it swells by a swelling ratio α to give a region of thickness $\delta_0 x\alpha$ (Fig. 1b). The thickness of the tablet is then given by

$$\begin{aligned}\delta &= \delta_0(1-x) + \alpha\delta_0 x \\ &= \delta_0(1 + x(\alpha - 1))\end{aligned} \quad (1)$$

Eqn. 1 shows that $\alpha < 1$ results in shrinkage.

Let the rate of penetration of water be inversely proportional to the thickness of the swollen layer. One mechanism is capillary suction at the dry/wet region interface, being instantaneously balanced by Darcy flow through

the swollen layer of thickness L;

$$\Delta P \sim \gamma\kappa = \mu L u^* k \tag{2}$$

where μ is the dynamic viscosity of water, γ the surface tension, κ the liquid curvature in the pores, u^* the superficial velocity of liquid through the swollen layer, and k its permeability. Rearranging Eqn. 2, and noting that u^* is related to the flux of solvent and thus the rate of swelling gives

$$-\frac{\mathrm{d}}{\mathrm{d}t}(\delta_0(1-x)) = +\delta_0\frac{\mathrm{d}\,x}{\mathrm{d}\,t} = \frac{K}{\alpha\delta_0 x} \tag{3}$$

$$\frac{\mathrm{d}x}{\mathrm{d}t} = \frac{K}{\alpha\delta_0^2 x} \tag{4}$$

where K is determined by permeability and pore suction. Integration with respect to x and t gives

$$\frac{\alpha\delta_0^2 x^2}{2} = Kt \tag{5}$$

Rearranging for x results in

$$x = \sqrt{\frac{2Kt}{\alpha\delta_0^2}} \tag{6}$$

The change in tablet thickness with time is given by substituting Eqn. 6 into Eqn. 1 to give

$$\begin{aligned}\frac{\delta}{\delta_0} &= 1 + (\alpha-1)\left(\frac{2Kt}{\alpha\delta_0^2}\right)^{0.5} \\ &= 1 + \left[\frac{2K(\alpha-1)^2}{\alpha\delta_0^2}\right]^{0.5} t^{0.5}.\end{aligned} \tag{7}$$

Hence the penetration front will propagate as a function of the square root of time.

II. VALIDATION OF THE MODEL

The validity of the permeation model can be supported by comparison with estimates of quantities using results from transport through packed beds. The biopolymer fibres are again assumed not to change volume.

Consider a 1.5 mm thick tablet of initial porosity 0.10 which swells by 0.3 mm in 24 s (Fig. 5a of the main manuscript). Assuming that the whole tablet has reached its equilibrium swelling thickness after 24 s,[3] the swollen voidage, ε, can be estimated by a volume balance on the solids,[4] viz.

$$\begin{aligned}\delta_0(1-\varepsilon_0) &= \alpha\delta_0(1-\varepsilon) \\ 1.5(1-0.10) &= \frac{2.1}{1.5}\cdot 1.5(1-\varepsilon) \\ \varepsilon &= 0.36\end{aligned}$$

where ε_0 is the initial voidage of the tablet.

The rate of flow through a packed bed of particles can be estimated using the Carman-Kozeny model,

$$\Delta P = \frac{180\cdot\mu\cdot u^*(1-\varepsilon)^2 L}{D_s^2\varepsilon^3}$$

Taking the size of the average MCC particle to be 25 μm,[5] and a value of $\mu = 1\times10^{-3}$ Pa s gives

$$\begin{aligned}\Delta P &= \frac{180\cdot 10^{-3}\cdot u^*\cdot(0.64)^2\cdot L}{(25\times10^{-6})^2\cdot 0.36^3} \\ &= \underbrace{2.6\times10^9}_{\text{Parameter } a}\cdot u^*\cdot L\end{aligned} \tag{8}$$

Let the estimate of the curvature in the pores be of the order of $2/D_s$.[5] Using Eqn. 2 and a value of $\gamma = 72\,\mathrm{mNm^{-1}}$ this gives

$$\begin{aligned}\Delta P &\sim 0.072\cdot\frac{2}{25\times10^{-6}} \\ &\sim 4.3\times10^3\,\mathrm{Pa}\end{aligned}$$

Using Eqn. 8 this gives

$$u^* = \frac{1.7\times10^{-6}}{L}$$

Relating u^* to Eqn. 3 requires a volume balance: per unit of cross-sectional area the flow must provide the extra water (solvent) to create the swollen region:

$$\begin{aligned}\underbrace{u^*\mathrm{d}\,t}_{\text{Flow in}} &= \underbrace{\mathrm{d}(\delta_0 x\alpha\varepsilon - \delta_0 x\varepsilon_0)}_{\text{Change in volume}} \\ &= \mathrm{d}(\delta_0 x)[\alpha\varepsilon - \varepsilon_0]\end{aligned}$$

$$\text{Giving}\quad \frac{\mathrm{d}\,(\delta_0 x)}{\mathrm{d}\,t} = \frac{u^*}{(\alpha\varepsilon-\varepsilon_0)} = \frac{\Delta P}{aL(\alpha\varepsilon-\varepsilon_0)}$$

Rearranging into the form of Eqn. 3 results in

$$K = \frac{\Delta P}{a(\alpha\varepsilon-\varepsilon_0)}$$

Using the quantities determined for the parameters we get

$$-\frac{\mathrm{d}}{\mathrm{d}t}(\delta_0(1-x)) = \underbrace{\frac{4.3\times10^3}{2.6\times10^9\cdot(\alpha\varepsilon-\varepsilon_0)}}_{K}\frac{1}{\alpha\delta_0 x}$$

$$K = \frac{1.7\times10^{-6}}{\frac{2.1}{1.5}0.36-0.1} = 4.1\times10^{-6}$$

Substituting this value of K into Eqn. 7 results in

$$\begin{aligned}\frac{\delta}{\delta_0} &= 1 + \left[\frac{2\cdot 4.1\times 10^{-6}(\alpha-1)^2}{\alpha\delta_0^2}\right]^{0.5} t^{0.5} \\ &= 1 + \left[\frac{2\cdot 4.1\times 10^{-6}\cdot 0.4^2}{1.4\cdot(1.5\times 10^{-3})^2}\right]^{0.5} t^{0.5} \\ &= 1 + 0.65\, t^{0.5}\end{aligned}$$

For $t = 24\,\text{s}$, the expression becomes $\delta = 1.5\,\text{mm}\cdot(1 + 0.65\cdot 4.90) = 6.3\,\text{mm}$. The value obtained from this model is significantly larger compared to the actual thickness of the swollen tablet (2.1 mm). However, it is overall consistent with the experimental data and the results are of the same order of magnitude. Given that this simple model is based on very basic assumptions with regards to the transport in the MCC matrix it is clear that a proper description of the transport processes in such immediate release formulations will require refined models.

It is important to keep in mind that the Carman-Kozeny expression for pressure drop in a packed bed is well known to over-predict the permeability in real powder systems.[5] If one were to assume a value for $K = 1\times 10^{-7}$, a change in K by a little more than one order of magnitude, well in line with previous experimental observations, the resultant thickness of the swollen polymer would be $\delta = 1.5\,\text{mm}\cdot(1 + 0.2\cdot 4.90) = 3.0\,\text{mm}$, a value much closer to the experimentally observed thickness δ.

As outlined above, the model does not describe the experimental results perfectly. Erosion can take place at the surface of the tablet where the solvent penetration occurs due to the laminar flow of the liquid at the back and the mechanical instability of the hydrated polymer. This might accelerate the liquid ingress and swelling compared to the model. Based on our assumption that the expansion of the tablet towards the penetrating liquid is of the same magnitude as the swelling of the dry side of the opposite face, which is the experimentally measured parameter, a swelling profile can be extracted (Fig. 2).

Overall the experimental data appears to follow the same kinetics compared to the model however the actual swelling is much slower compared to the model prediction. This result is not surprising given that the transport model does not take into account any effects due to the change in viscosity as soon as the MCC matrix becomes hydrated as well as the gradual changes in voidage that will take place as the polymer matrix hydrates. Upon adjusting the value of K within the limits of previously reported range suitable to account for the over-estimation typically observed for the Carman-Kozeny model a very good qualitative agreement can be demonstrated between the model and the experimental data.

Based on the model it is possible to estimate the effect of changes in temperature on the permeation (Fig. 3).

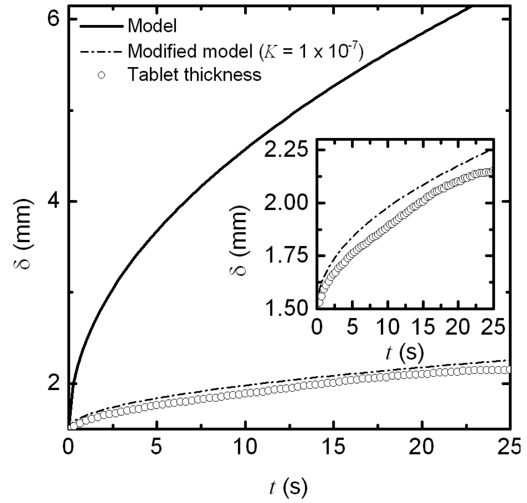

FIG. 2. Comparison between the predicted swelling kinetics using the permeation model compared to the experimental data. The inset shows a more detailed comparison between the experimental data and the model using the adjusted parameter K

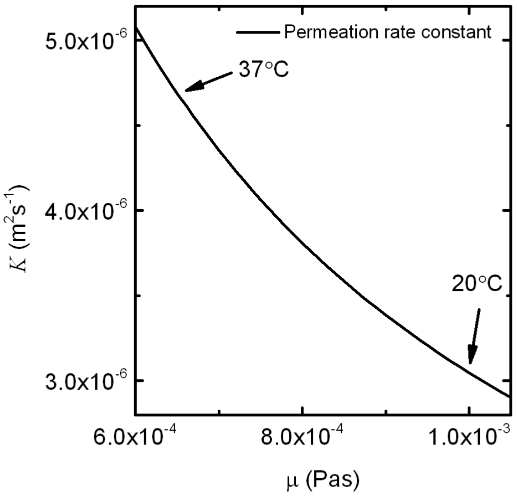

FIG. 3. Effect of the change in viscosity on the Permeation rate constant K. Upon increasing the temperature from 20 to 37°C the permeability increases significantly.

III. CONCLUSIONS

Even though the model is derived for a tablet composed from a single excipient its general form is valid also for multicomponent tablets. However, in order to extract meaningful physical parameters the model has to be adjusted in terms of the particle size distributions and the corresponding volume fractions of the respective excipients to obtain the correct value of K. Overall the liquid front will propagate as a function of the square root over time.

NOMENCLATURE

α	Swelling ratio
δ	Tablet thickness at time t (m)
δ_0	Dry thickness of tablet (m)
γ	Surface tension (Nm^{-1})
κ	Liquid curvature in the pore
μ	Dynamic viscosity (Pa·s)
ε	Swollen voidage
ε_0	Porosity or voidage
D_s	Particle diameter (m)
K	Permeation rate constant (m^2s^{-1})
k	Permeability (m^{-2})
L	Thickness of swollen layer $\equiv \delta_0 x\alpha$ (m)
P	Pressure (Pa)
u^*	Superficial velocity of liquid through the swollen layer (ms^{-1})
x	Function of material contacted with water

* jaz22@cam.ac.uk; http://thz.ceb.cam.ac.uk

[1] D. G. Fredlund and V. Dakshanamurthy, Geochemical Engineering **13**, 15 (1982).

[2] A. Szymkiewicz, in *Modelling Water Flow in Unsaturated Porous Media* (Springer Berlin Heidelberg, Berlin, Heidelberg, 2013) pp. 9–47.

[3] It is important to note that Eqn. 7 does not predict equilibrium swelling and hence this assumption is based on a inherent simplification of the model.

[4] As outlined earlier we assume that the swelling occurs uniformly in both directions and hence the magnitude of swelling measured by TPI (towards the terahertz optics) is matched by swelling of the back surface by an equal amount. The total swelling is therefore 0.6 mm.

[5] S. Mascia, *Rheology and processing of pharmaceutical pastes*, Ph.D. thesis, Cambridge, UK (2008).
